# Mesenteric tissue for the treatment of septic pelvic complications in the absence of greater omentum

**DOI:** 10.1007/s10151-016-1549-9

**Published:** 2016-12-02

**Authors:** E. J. de Groof, O. van Ruler, C. J. Buskens, P. J. Tanis, W. A. Bemelman

**Affiliations:** Department of Surgery, Academic Medical Center, PO BOX 22660, 1100 DD Amsterdam, The Netherlands

## Introduction

A presacral abscess or sinus is a potentially devastating complication. These may result from an infectious disease or post-operative complications such as anastomotic leakage. A persisting presacral sinus may lead to fistula formation [1, 2]. Salvage surgery may be indicated, and an omentoplasty or myocutaneous flap reconstruction can be used to fill dead space and control local pelvic sepsis [3]. Greater omentum is not always available, and tissue flaps have the risk of flap necrosis. We describe four cases in which mesenteric tissue surrounding either branches of the inferior mesenteric or ileocolic artery was used to fill the pelvis.

## Technique

**Fig. 1:**
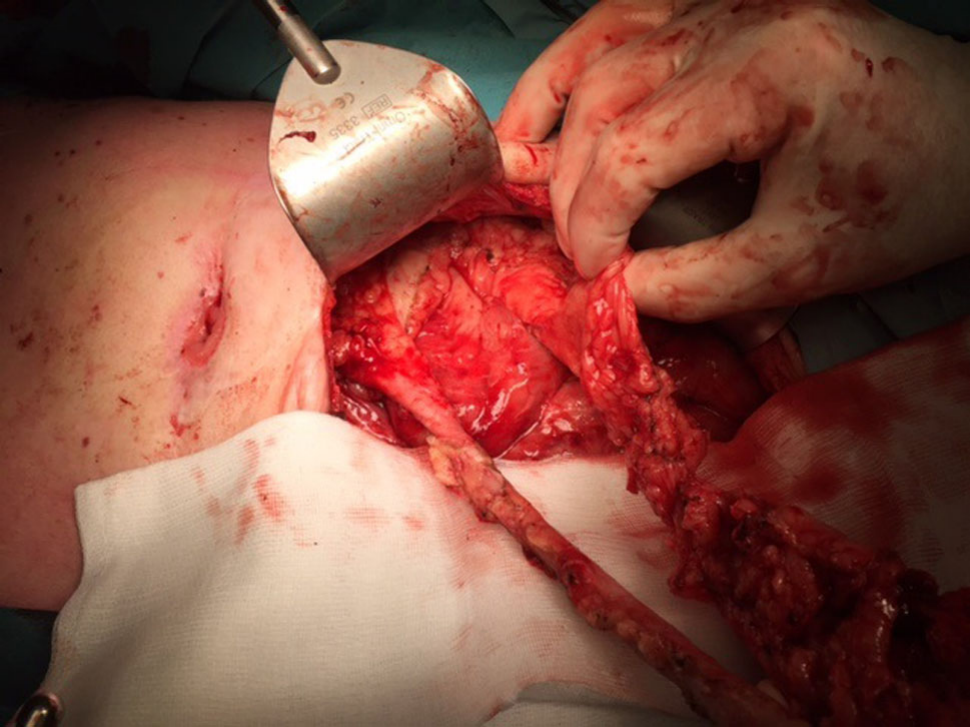
Male patient (69 years old) with persistent leakage of the coloanal anastomosis treated with resection of the efferent loop of the diverting colostomy and rectal stump with debridement of a presacral abscess. The mesentery was fully mobilised and moved towards the dead space in the pelvic cavity

All patients had a pre-existing deviating ileostomy or colostomy. To resect the remaining rectum or ileal pouch-anal anastomosis, a transanal intersphincteric approach was used with thorough debridement of the presacral sinus/abscess. There was not enough omentum to create an omentoplasty of sufficient length and volume. The colon or ileum was dissected close to the bowel, thereby leaving the recto-sigmoid mesentery or ileocecal mesentery in situ with its vascular supply. Mesentery was fully mobilised and moved towards the pelvic dead space (Fig. 1). Fixation to the pelvic wall and/or pubic bone was performed to prevent small bowel loop herniation. Pelvic drains were placed.

## Results

Baseline patient characteristics are displayed in Table 1. In one patient, resection of a coloanal anastomosis was performed for persistent leakage, with a history of iatrogenic rectal perforation after cystoprostatectomy. Another patient had a persistent presacral sinus due to fistulisation from an ileal pouch-anal anastomosis. The third patient also had an ileal pouch-anal anastomosis for ulcerative colitis, but was rediagnosed with Crohn’s disease. Indications for pouch excision were persisting pouchitis and cuffitis with perianal fistulas. The fourth patient had a history of cystoprostatectomy and a Hartmann’s procedure, complicated by recurrent abscess and fistula formation from the rectal stump, for which a coloanal reconstruction with diverting colostomy and multiple endosponge procedures were performed.

**Table 1:** Baseline characteristics of included patients

Baseline characteristics	Patient 1	Patient 2	Patient 3	Patient 4
Sex	Male	Male	Male	Male
Age at surgery (years)	74	55	44	69
BMI (kg/m^2^)	28.1	26.6	22.1	21.0
ASA classification	2	2	3	2
Diagnosis	Bladder cancer	Ulcerative colitis	Crohn’s disease	Bladder cancer
Previous (abdominal and/or pelvic) surgery	Cystoprostatectomy complicated by rectal perforation treated with Hartmann’s procedure (’11)	Perforated colon treated with subtotal colectomy + ileostomy, second-stage completion proctectomy + ileo-pouch-anal anastomosis (’03)	Toxic megacolon treated with subtotal colectomy, complicated by idiopathic thrombocytopenic purpura (‘11)	Cystoprostatectomy (’96), complicated by abscess + fistulas
	Coloanal pouch + loop colostomy + Ramirez plasty + bridging biomesh, complicated by anastomotic leakage treated with endosponge (’13)	Perianal fistulas + pouchitis treated with loop ileostomy + fistula drainage (’15)	Completion proctectomy + ileal –pouch-anal anastomosis + ileostomy + splenectomy, complicated by bleeding treated with relaparotomy + coiling inferior mesenteric artery (’12)	Hartmann’s procedure (‘03) with multiple stoma revisions + endosponge (‘05)
			Presacral haematoma treated with relaparotomy + secondary closure abdomen with mesh (’12)	Coloanal anastomosis + colostomy closure, complicated by anastomotic leakage with creation of double-loop transverse colostomy (’07)
			Ileal pouch-anal anastomosis dehiscence treated with endosponge, multiple transanal defect closures + pouch redo’s + Ramirez plasty (‘12–’15)	

*BMI* body mass index, *ASA* American Society of Anesthesiologists

Surgical details are presented in Table 2. The post-operative course was uneventful in one patient (Table 3). One patient developed a subhepatic abscess, which was punctured. The two remaining patients had persisting pelvic abscesses, treated by antibiotics in one patient, and, in the other, percutaneous drainage which failed necessitating surgical drainage. Eventually, all patients recovered without signs of pelvic infection.

**Table 2:** Surgical characteristics of included patients

Surgical characteristics	Patient 1	Patient 2	Patient 3	Patient 4
Indication	Persisting leakage coloanal anastomosis	Ileal pouch-anal anastomosis with persistent fistulas	Ileal pouch-anal anastomosis with persistent presacral sinus	Persistent leakage of coloanal anastomosis
Surgery	Resection efferent loop of diverting colostomy and rectal stump with debridement of pelvic abscess	Excision of ileal pouch-anal anastomosis with creation of end ileostomy	Excision of ileal pouch-anal anastomosis with creation of end ileostomy	Resection of efferent loop of diverting colostomy and rectal stump with debridement of presacral abscess
Approach	Laparotomy	Laparotomy	Laparotomy and transanal minimally invasive surgery	Laparotomy and transanal minimally invasive surgery
Setting	Elective	Elective	Elective	Elective
Blood loss (ml)	NR	400	100	100

*NR* not reported

**Table 3:** Post-operative outcomes of included patients

Post-operative outcomes	Patient 1	Patient 2	Patient 3	Patient 4
Post-operative stay (days)	19	6	25	16
Post-operative complications	Pelvic abscess	No	Subhepatic abscess and ileus	Small pelvic abscess
Reintervention	Percutaneous drainage	No	Diagnostic puncture and peripherally inserted central catheter for total parenteral nutrition	No
Readmission (within 30 days)	Yes	No	No	No
Late complications	Persistent pelvic abscess	No	Granuloma at stoma site	No
Follow-up to date (months)	22	4	4	1

## Discussion

Salvage surgery for pelvic septic complications following colorectal surgery most often dictates radical removal of pelvic bowel structures with a definitive ostomy [4]. Patients undergoing redo surgery are prone to develop recurrent infectious complications. Contaminated pelvic dead space after salvage surgery may progress into a sinus with persistent abscesses and the risk of secondary complications. Previous research suggests that obliterating the pelvic space with an omentoplasty after abdominoperineal resection for rectal cancer results in enhanced perineal wound healing and a decrease in sinus formation due to angiogenesis and enhancement of the inflammatory response [5]. Pelvic dead space obliteration after salvage surgery is also described for this purpose [4]. In the absence of omentum, and considering the morbidity associated with autologous tissue flaps, obliteration of pelvic dead space with viable mesentery of a bowel segment that has to be removed as part of salvage procedures seems to be a valuable alternative. Although one patient had a persistent pelvic abscess, complete pelvic sinus healing was accomplished in all four patients.

More research is necessary to understand the physiological immune responses of mesentery, which may be of value in controlling infectious complications not just for anatomical filling. Availability of mesenteric tissue of adequate length and volume has to be assessed in every single patient, but might be preferred over myocutaneous flap reconstructions.
